# Supplemental *Clostridium butyricum* MIYAIRI 588 Affects Intestinal Bacterial Composition of Finishing Pigs

**DOI:** 10.1264/jsme2.ME22011

**Published:** 2022-09-23

**Authors:** Maki Hirata, Miki Matsuoka, Takuma Hashimoto, Takamichi Oura, Yo Ohnuki, Chika Yoshida, Ayaka Minemura, Daiki Miura, Kentaro Oka, Motomichi Takahashi, Fumiki Morimatsu

**Affiliations:** 1 Bio-Innovation Research Center, Tokushima University, Tokushima, Japan; 2 Faculty of Bioscience and Bioindustry, Tokushima University, Tokushima, Japan; 3 R&D Division, Miyarisan Pharmaceutical Co., Ltd., Saitama, Japan

**Keywords:** *Clostridium butyricum*, pig, gut microbiota, probiotics

## Abstract

Animal gastrointestinal tracts are populated by highly diverse and complex microbiotas. The gut microbiota influences the bioavailability of dietary components and is closely associated with physiological processes in the host. *Clostridium butyricum* reportedly improves growth performance and affects the gut microbiota and immune functions in post-weaning piglets. However, the effects of *C. butyricum* on finishing pigs remain unclear. Therefore, we herein investigated the effects of *C. butyricum* MIYAIRI 588 (CBM588) on the gut microbiota of finishing pigs. 16S rRNA gene sequencing was performed using fecal samples and ileal, cecal, and colonic contents collected after slaughtering. The α-diversity of the small intestinal microbiota was lower than that of the large intestinal microbiota, whereas β-diversity showed different patterns depending on sample collection sites. The administration of CBM588 did not significantly affect the α- or β-diversity of the microbiotas of fecal and intestinal content samples regardless of the collection site. However, a linear discriminant ana­lysis Effect Size revealed that the relative abundance of *Lactobacillaceae* at the family level, *Bifidobacterium* at the order level, and *Lactobacillus ruminis* and *Bifidobacterium pseudolongum* at the species level were higher in the fecal samples and cecal and colonic contents of the treatment group than in those of the control group. Therefore, the administration of CBM588 to finishing pigs affected the composition of the gut microbiota and increased the abundance of bacteria that are beneficial to the host. These results provide important insights into the effects of probiotic administration on relatively stable gut microbial ecosystems.

A balanced bacterial flora in the gastrointestinal tract plays important physiological, neurological, and immunological roles in hosts (Lynch and Pedersen, 2016; Lazar *et al.*, 2018; Parker *et al.*, 2020). In pigs, feed additives, including probiotics, alter the gut microbiota and improve growth performance (Wei *et al.*, 2020; Liang *et al.*, 2021). Accordingly, interventions that influence the composition and activity of the gut microbiota have potentially important economic implications for pig farming (Sato *et al.*, 2019; Wang *et al.*, 2019).

The introduction of exogenous microbial communities may effectively confer disease resistance (Round and Mazmanian, 2009; Brestoff and Artis, 2013). Freshly weaned piglets are less resistant to disease than older pigs, and many studies have examined the intestinal microbiota (Luise *et al.*, 2021; Saladrigas-Garcia *et al.*, 2021). In contrast, limited information is currently available on the effects of probiotic administration to finishing pigs. Studies on the intestinal microbiota of pigs have attracted considerable attention due to their ability to serve as excellent biomedical models (Heinritz *et al.*, 2013). Therefore, studies on older pigs with a relatively stable microbiota (Torow and Hornef, 2017) provide important insights into the effects of gut microbiota modulations on host physiology.

Probiotic feed additives have gained widespread attention in the livestock industry since the European Union’s ban on antibiotic growth promoters. The functional mechanisms underlying the beneficial effects of probiotics were previously suggested to be multifactorial, including protection from pathogens in the gut and the stimulation of host immune responses. Among the bacteria used as probiotics, *Clostridium butyricum* is a butyrate-producing, Gram-positive anaerobe that is present in soil and also in the gut of healthy humans and animals. *C. butyricum* promotes the growth of other beneficial bacteria, such as *Bifidobacterium* spp. and *Lactobacillus* spp., and inhibits that of harmful bacteria, including *Escherichia coli*, thereby contributing to a favorable balance in the intestinal environment (Kong *et al.*, 2011). Consequently, *C. butyricum* is widely used as a probiotic or live biotherapeutic product in human pharmaceuticals. *C. butyricum* strain MIYAIRI 588 (CBM588) is used as a feed additive to improve the health and growth performance of livestock (Takahashi *et al.*, 2018) and to treat digestive disorders in humans (Seki *et al.*, 2003). Although the effects of *C. butyricum* on the gut microbiota of post-weaning piglets have been examined, its impact in growing pigs has yet to be clarified.

Therefore, we herein investigated the effects of dietary supplementation with CBM588 on the intestinal bacterial composition of finishing pigs. By examining changes in the gut microbiota with the administration of CBM588 to pigs, we assessed the effects of probiotic addition on a relatively stable gut ecosystem. The present results will contribute to a more detailed understanding of the impact of specific feeding practices on the bacterial ecosystem in the gastrointestinal tract of pigs.

## Materials and Methods

The protocols used for animal experimental procedures were approved by the Institutional Animal Care and Use Committee of Tokushima University (approval number: T2019-112). All experimental animals were housed in a pig research facility (Advanced Livestock System Center) at Tokushima University.

### Animals and feeding

Crossbred pigs were housed individually in an environmentally controlled room under standard management. Pig health and fecal characteristics were monitored daily. The basal feed was free of intestinal microbiota modifiers, such as antimicrobials and probiotics. Twenty pigs, comprising a mixture of castrates and females aged 88 days at the start of the study (the offspring of three sows), were obtained from a local commercial farm and offered feed and water *ad libitum*. The initial body weight of the pigs before the experimental period was approximately 40 kg. Animals were subjected to a preliminary 2-week acclimatization phase before the start of the experiments. Pigs were divided into two groups based on litter origin followed by body weight and then sex. Pigs in the control group were fed a basal diet, whereas those in the treatment group were fed a basal diet supplemented with CBM588 at 2.5×10^8^‍ ‍CFU‍ ‍kg^–1^ feed. We confirmed that each group had similar mean weights at the start of the study and similar weight gain and no infectious diseases during the experimental period.

### Sample collection, DNA purification, 16S rRNA gene sequencing, and real-time PCR

Pigs were slaughtered at 5 months of age, and the contents of three intestinal segments (the ileum, cecum, and colon) were collected. Fecal samples were collected 4 days before slaughter. The collected samples of intestinal contents and feces (approximately 100‍ ‍mg) were suspended in 900‍ ‍μL of guanidine thiocyanate solution (4 M guanidine thiocyanate, 100‍ ‍mM Tris-HCl [pH 9.0], and 40‍ ‍mM EDTA). Regarding extraction, 80–100‍ ‍μL of the suspension was diluted in TE buffer to a final volume of 600‍ ‍μL, to which glass beads (0.3 g, diameter of 0.15–0.21‍ ‍mm) were added, followed by beating with a MagNA Lyser (Roche) operating at 7,000‍ ‍rpm for 20 s. Thereafter, 600‍ ‍μL of phenol/chloroform/isoamyl alcohol (25:24:1 [v/v/v]) and 100‍ ‍μL of 10% sodium dodecyl sulfate (w/v) were added, and bead fractionation was repeated. The resulting suspension was then incubated at 70°C for 10‍ ‍min before a final round of bead fractionation. The samples obtained were centrifuged at 20,800×*g* at room temperature for 5‍ ‍min, and the resulting upper layer was transferred to 1.5-mL centrifuge tubes. After the addition of 600 μL of ice-cold isopropanol and 60 μL of 3 M sodium acetate, the tubes were inverted several times to mix the contents. The tubes were then centrifuged at 20,800×*g* for 5‍ ‍min, and the resulting supernatants were discarded by decanting. The remaining DNA pellets were washed with 70% ice-cold ethanol and dried in a centrifuge evaporator (Eyela) for 5‍ ‍min. Thereafter, the pellets were dissolved in 200‍ ‍μL of TE, and a High Pure PCR template preparation kit (Roche) was used for further DNA purification. The V3–V4 region of the 16S rRNA gene was amplified via a polymerase chain reaction (PCR) using barcoded primers, as previously reported (Hagihara *et al.*, 2020). The KOD One PCR Master Mix (TOYOBO) was used for PCR amplification, and the resulting PCR products were purified based on size selection using SPRI select (Beckman Coulter). DNA was quantified using a Qubit dsDNA BR assay kit (Thermo Fisher Scientific). Mixed samples were prepared by pooling approximately equal amounts of amplified and purified DNA, and these were sequenced using MiSeq Reagent Kit V3 (600 cycles) and a MiSeq sequencer (Illumina) according to the manufacturer’s instructions. In the sequence ana­lysis, 16S rRNA gene sequence data obtained from the MiSeq sequencer were processed using Quantitative Insights into Microbial Ecology (QIIME 2 2019. 10, http://qiime2.org/) (Bolyen *et al.*, 2019). An amplicon sequence variant (ASV) table was obtained by the demultiplexing and quality filtering of raw sequence data with the q2-demux plugin, followed by denoising with DADA2 (Callahan *et al.*, 2016). The taxonomy of each variant was assigned at the species level by comparisons with the SILVA database (Quast *et al.*, 2013). Within-community diversity (α-diversity) was examined using the Shannon index, the values of which were calculated using QIIME2. To assess distances between samples, β-diversity was estimated using the weighted UniFrac metric and visualized using a principal coordinate ana­lysis (PCoA). A linear discriminant ana­lysis (LDA) Effect Size (LEfSe) (Segata *et al.*, 2011) with default settings, except for the threshold on the logarithmic LDA score, which was set as 3.0, was used to identify bacterial features differentially represented between the control and treatment groups.

### Real-time PCR

Quantitative real-time PCR was performed to estimate the cell numbers of total bacteria and *C. butyricum* based on DNA samples purified from cecal and colonic digesta using TB Green Premix EX Taq II (Tli RNase H Plus, Takara Bio), as described in a previous study (Tomita *et al.*, 2022). To create a standard curve, the number of CBM588 was counted using a phase-contrast microscope. Bacterial quantity was calculated from Ct values based on standard curves obtained by the serial dilution of DNA extracted from cultured CBM588. Real-time PCR was performed using StepOnePlus (Thermo Fisher Scientific). The following primer sets described in previous studies were used (Tomita *et al.*, 2022): total bacteria, 5′-CGGYCCAGACTCCTACGGG-3′ and 5′-TTACCGCGGCTGCTGGCAC-3′, *C. butyricum*, 5′-AGTGATTGTCAGTAGTAGACGAGCG-3′ and 5′-CATGCGCCCT TTGTAGC-3′. The amplification program included an initial denaturation step at 95°C for 30‍ ‍s followed by 40 cycles of denaturation at 95°C for 5‍ ‍s, and annealing/extension at 60 or 60.5°C for total bacteria or *C. butyricum*, respectively, for 30 s. The specificity of the amplified products was confirmed using the melting temperature and dissociation curve after amplification. The relative abundance of *C. butyricum* was calculated by dividing the cell number of *C. butyricum* by the total number of bacterial cells.

### Statistical ana­lysis

The Kruskal–Wallis test was used to compare the Shannon diversity index value for the ileal, cecal, and colonic contents and fecal samples obtained from the control and treatment groups. A cut-off FDR of 0.05 was applied based on the Benjamini–Hochberg (BH) method. Comparisons of β-diversity indices were calculated using a permutational multivariate ana­lysis of variance (PERMANOVA). The Mann–Whitney U test was used to assess the significance of differences in the abundance of gut microbiota between the control and treatment groups. Differences of *P*≤0.05 were considered to be significant.

## Results

### α- and β-Diversities of the gut microbiota

**Fig. 1.**
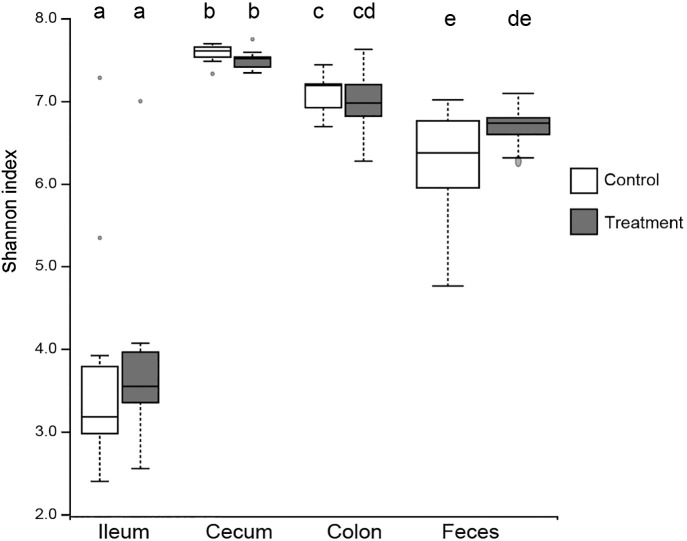
α-Diversity of bacteria in gut contents of finishing pigs. Box plots show diversity (Shannon index) based on amplicon sequence variant (ASV) abundance in the contents of the ileum, cecum, and colon of pigs collected after slaughter. In each boxplot, *n*=10. FDR adjusted p-values are from the Kruskal-Wallis test. Different letters indicate a significant difference (*P*<0.05).

**Fig. 2.**
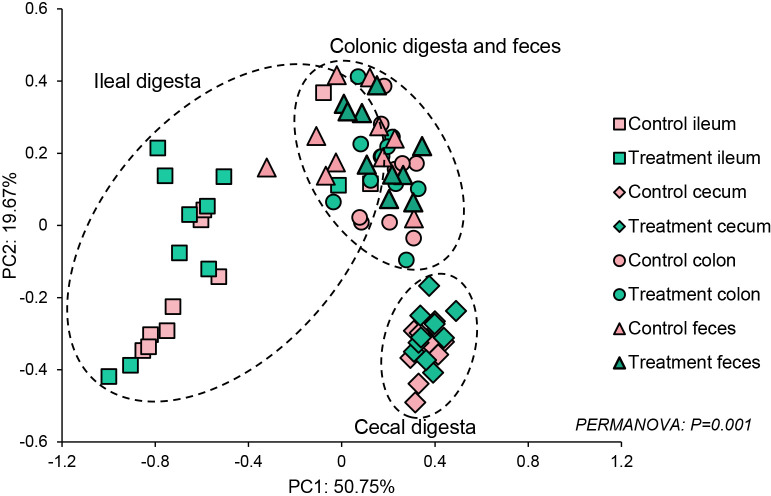
Principal coordinate ana­lysis (PCoA) plots of gut microbiota clustering. PCoA plots were based on weighted UniFrac distances of the gut contents of the control and CBM588-treated groups. Each dot represents an individual sample.

Irrespective of the intestinal segment, no significant differences were observed in terms of the α-diversity of the intestinal digesta and fecal samples based on the Shannon index between the control and treatment groups (Fig. 1). However, Shannon indices significantly differed among the ileal, cecal, and colonic digests and fecal samples of both groups, except for between the colonic digests and fecal samples of the treatment group. (Fig. 1, *P*<0.05). Similarly, a significant shift in β-diversity was detected among the intestinal segments from which the digesta had been collected (Fig. 2). The fecal and colonic samples of the control and treatment groups formed close clusters, whereas the β-diversities of the colonic digesta and fecal samples of the control group significantly differed (Fig. 2, *P*<0.05). No significant differences were observed in the β-diversities of the three intestinal segments and fecal samples between the control and treatment groups.

### Abundance of the gut microbiota

**Fig. 3.**
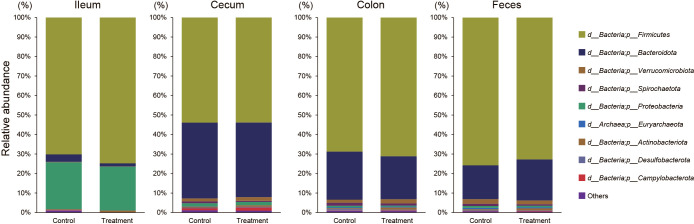
Composition of the gut microbiota of finishing pigs at the phylum level. The relative abundance of bacteria in the gut contents of the control and CBM588-treated groups. The values used to compose the figures represent group mean relative abundances.

Analyses of the microbial composition revealed that *Firmicutes* and *Bacteroidetes* were the two most abun­dant‍ ‍bacterial phyla in cecal and colonic digesta and fecal samples (Fig. 3 and Table 1). However, distinct profiles were observed in the ileum, in which *Firmicutes* and *Proteobacteria* predominated. Other general observations at the phylum level included *Verrucomicrobia*, *Spirochaetes*, and *Proteobacteria* were the three phyla followed by the‍ ‍next two phyla in the colonic digesta of the control group, whereas *Actinobacteria* was more abundant than *Proteobacteria* in the treatment group. The percentage of *Actinobacteria* in the cecal and colonic digesta was significantly higher in the treatment group than in the control group (Table 1, *P*<0.05). In contrast, no significant differences were noted in bacterial phyla between the ileal digesta of the control and treatment groups.

**Fig. 4.**
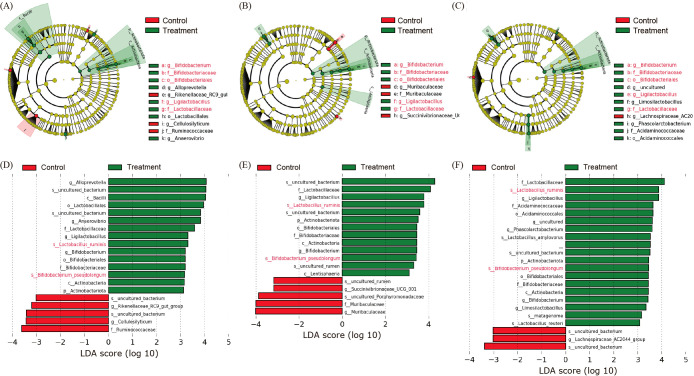
Taxonomic differences between control and CBM588-treated groups. A cladogram showing taxonomic differences between the control and treatment groups with respect to (A) feces, (B) the colon, and (C) cecum. Linear discriminant ana­lysis (LDA) Effect Size results for (D) feces, (E) the colon, and (F) cecum at the species level (LDA score >3.0). Taxa with a higher abundance in the cecal and colonic digesta and feces of the CBM588-treated group are shown with text in red.

Similar differences were detected in cecal and colonic digesta and fecal samples at the phylum level between the control and treatment groups after the administration of CBM588. Therefore, we focused on these samples and performed a LEfSe ana­lysis to examine the effects of CBM588 on the composition of the gut microbiota at the species level. The results obtained revealed that the relative abundance of *Lactobacillaceae* at the family level, *Bifidobacterium* at the order level (Fig. 4A, B, and C), and *Lactobacillus ruminis* and *Bifidobacterium pseudolongum* at the species level were consistently higher in the cecal and colonic digesta and fecal samples of the treatment group than in those of the control group (Fig. 4D, E, and F). The average relative abundance of *C. butyricum* in the cecal and colonic digesta was higher after the administration of CMB588 than that in the control group; however, the difference between the control and treatment groups was not significant (Supplemental Table S1). To further investigate differences in the abundance of *C. butyricum* in cecal and colonic digesta, real-time PCR was performed using intergenic spacer region (ISR)-type B-specific primers to analyze CBM588-containing *C. butyricum* strains (Nakanishi *et al.*, 2005; Qadis *et al.*, 2014). The detection rates of *C. butyricum* in the cecal and colonic digesta of the control group were 0% (0/10) and 10% (1/10), respectively, with a relative abundance of 2.1×10^–5^% in the colonic digesta. In contrast, the detection rates of *C. butyricum* in the cecal and colonic digesta of the treatment group were 100% (10/10) and 90% (9/10), respectively, with average relative abundance ratios of 4.4×10^–5^±7.2×10^–6^ and 4.3×10^–5^±5.9×10^–6^%, respectively.

## Discussion

In the present study, we examined the effects of dietary supplementation with *C. butyricum* strain MIYAIRI 588 (CBM588) on the intestinal microbiota of finishing pigs. The results obtained revealed that *Firmicutes* and *Bacteroidetes* were the two most abundant bacterial groups at the phylum level in control pigs, which is consistent with previous findings on the intestinal microbiota of finishing pigs (McCormack *et al.*, 2017; Sato *et al.*, 2019; Bunte *et al.*, 2020). Other studies reported different proportions in the gut microbiota, with *Actinobacteria* or *Proteobacteria* being more abundant that *Bacteroidetes* in colonic contents during the growth period (Xiao *et al.*, 2018; Ruczizka *et al.*, 2019). The gut microbiota is considered to be influenced by a diverse range of factors, including breed, age, and diet (Claesson *et al.*, 2011; Xiao *et al.*, 2018; Rinninella *et al.*, 2019; Pu *et al.*, 2020). Moreover, the vertical transfer of the intestinal microbiota across generations has been reported, and changes in its composition due to dietary differences were found to be magnified across generations in mice (Sonnenburg *et al.*, 2016). Accordingly, differences among studies regarding the composition of the gut microbiota at the phylum level may be attributed to differences in the genetic background and/or environment of the pigs examined.

Previous studies demonstrated that the composition of the bacterial community in the intestinal tract of pigs was spatially heterogeneous (Looft *et al.*, 2014; Zhao *et al.*, 2015; Xiao *et al.*, 2018; Zhang *et al.*, 2018). Consistent with these findings, we showed that the microbiota of the ileal digesta exhibited lower diversity than that of the lower intestinal digesta (Zhang *et al.*, 2016). Furthermore, small variations in microbial compositions were observed between the cecal and colonic digesta and fecal samples; marked differences between the ileal digesta and lower gastrointestinal tract, including the cecum and colon, were consistent with previous findings (Zhao *et al.*, 2015; Zhang *et al.*, 2016). The shorter transit time, higher oxygen concentration, and lower pH in the ileum than in the lower gastrointestinal tract may make it a hostile environment for microorganisms to survive, and may contribute to the differences detected in the composition of the microbiota between the upper and lower gastrointestinal tracts (Donaldson *et al.*, 2016; Zhang *et al.*, 2018). In addition to environmental differences, the small and large intestines have functional differences; the small intestine is primarily responsible for digestion and absorption, whereas the large intestine is the site of microbial fermentation for the degradation of complex carbohydrates (Walter and Ley, 2011; Martinez-Guryn *et al.*, 2019). Therefore, the specificity of the microbial community may also be related to the function of each intestinal segment.

In the present study, we found that the administration of CBM588 to finishing pigs increased the abundance of *Actinobacteria* within the gut microbiota, which is consistent with previous findings showing that the abundance of *Actinobacteria* was increased by feeding CMB588 to post-weaning piglets (Sato *et al.*, 2019) and *C. butyricum* to pregnant sows (Cao *et al.*, 2019). The results of 16S rRNA gene sequencing revealed a slight increase in the abundance of *C. butyricum* in the cecal and colonic digesta after the administration of CBM588. Real-time PCR results further confirmed that the abundance of *C. butyricum* ISR-type B strains, including CBM588, slightly increased in the cecal and colonic digesta of the treatment group. Collectively, these results indicate that the dietary administration of CBM588 increased the abundance of *Actinobacteria* within the gut microbiota of pigs regardless of the growth stage. However, the administration of CBM588 did not affect the‍ ‍α- or β-diversity of the gut microbiota. The administration of CBM588 to pigs after weaning was previously shown to alter β-diversity and the abundance ratios of many phyla, families, and genera (Sato *et al.*, 2019). Conversely, the increase in *Bifidobacterium* after the administration of CBM588 in the present study was not detected in weaned piglets (Sato *et al.*, 2019). The dose of CBM588 in the study‍ ‍by Sato *et al.* was 9.6×10^7^ CFU kg^–1^, which was less than half of the dose administered in the present study (2.5×10^8^‍ ‍CFU‍ ‍kg^–1^). Different doses may have varying‍ ‍ef­fects on the intestinal microbiota. Furthermore, the gut microbiota dynamically changes during the weaning period (Slifierz *et al.*, 2015; Chen *et al.*, 2017), and the administration of CBM588 may have induced a stronger change in the gut microbiota than in fattening pigs. In addition, a previous study on the effects of CBM588 on the growth performance of post-weaning piglets reported discordant findings among studies, which may be attributed to differences in the breed, rearing environment, and feed (Takahashi *et al.*, 2018). Although the reasons for these heterogeneous results remain unclear, changes in the gut microbiota after the administration of CBM588 may differ depending on the growth stage or rearing of pigs and the study environment.

Our examination on the effects of CMB588 on the gut microbiota of finishing pigs at the species level revealed an increase in the abundance of *B. pseudolongum* and *L. ruminis*. Previous studies detected *B. pseudolongum* in the feces of humans, mice, and lactating piglets (Mikkelsen *et al.*, 2003; Turroni *et al.*, 2009; Mongodin *et al.*, 2017). In rats, the administration of *B. pseudolongum* strain Patronus reduced the population of *Akkermansia muciniphila* and increased the thickness of the submucosal muscular layer of the colon (Mangin *et al.*, 2018). Furthermore, in a mouse cancer model, the administration of *B. pseudolongum* activated antitumor T cells via metabolites, suggesting that *B. pseudolongum* increases the effectiveness of immunotherapy (Mager *et al.*, 2020). In contrast, *L. ruminis* has been isolated from both humans and pigs and has been suggested to affect host immune functions by activating TNF and producing IL-8 (Taweechotipatr *et al.*, 2009; Neville *et al.*, 2012). In the present study, the administration of CBM588 increased the abundance of *B. pseudolongum* and *L. ruminis*, which have been suggested to exert beneficial effects on host animals. Further studies are needed to establish whether the administration of CBM588 positively contributes to the health of pigs.

Orally administered CBM588 has been shown to promote an increase in the host intestinal flora, such as *Lactobacillus* and *Bifidobacterium* (Kong *et al.*, 2011; Hagihara *et al.*, 2021; Dizman *et al.*, 2022). Similar results were obtained in the present study. We also detected an increase in the abundance of two species upon the administration of CBM588; however, the underlying mechanisms remain unclear. CBM588 mainly produces butyric acid, which maintains a constant intestinal pH and, thus, inhibits the growth of harmful bacteria (Takahashi *et al.*, 2004). A CBM588 culture supernatant promoted the proliferation of *Bifidobacterium in vitro* (unpublished data), suggesting that some metabolites produced by CBM588 promote the growth of *Lactobacillus* and *Bifidobacterium*, including *B. pseudolongum* and *L. ruminis*. However, further studies are needed to elucidate the underlying mechanisms.

In conclusion, the dietary administration of CBM588 specifically increased the abundance of two bacterial species that may play a role in host immune functions. The potential for CBM588 to exert beneficial effects on host health via changes in the gut microbiota warrants further study.

## Citation

Hirata, M., Matsuoka, M., Hashimoto, T., Oura, T., Ohnuki, Y., Yoshida, C., et al. (2022) Supplemental *Clostridium butyricum* MIYAIRI 588 Affects Intestinal Bacterial Composition of Finishing Pigs. *Microbes Environ ***37**: ME22011.

https://doi.org/10.1264/jsme2.ME22011

## Supplementary Material

Supplementary Material

## Figures and Tables

**Table 1.** Relative abundance (%) of major bacterial phyla^1,2^

Phylum	Ileum			Cecum			Colon			Feces	
	Control	Treatment		Control	Treatment		Control	Treatment		Control	Treatment
*Firmicutes*	70.96±6.80	73.61±8.50		53.95±0.78	53.74±0.99		68.68±1.21	71.00±1.38		75.86±2.10	72.85±1.43
*Bacteroidetes*	4.23±2.29	1.89±1.81		38.77±0.86	38.35±0.88		24.75±1.05	22.20±1.27		17.27±2.16	20.93±1.56
*Verrucomicrobia*	0.20±0.14	0.11±0.10		1.54±0.19	1.87±0.20		1.53±0.19	2.12±0.36		2.40±0.33	1.94±0.28
*Spirochaetes*	0.09±0.08	0.11±0.11		0.97±0.33	0.58±0.16		1.55±0.34	1.10±0.18		1.10±0.24	0.82±0.17
*Proteobacteria*	22.58±7.02	23.16±8.85		1.56±0.20	1.67±0.30		0.85±0.17	0.60±0.08		0.99±0.19	0.76±0.14
*Actinobacteriota*	0.52±0.18	0.91±0.43		0.54±0.06 ^a^	0.81±0.09 ^b^		0.50±0.08 ^a^	1.10±0.15 ^b^		0.59±0.09 ^a^	1.12±0.16 ^b^

^1^ The top six bacterial phyla found in fecal samples of the control group are shown. Data are expressed as the mean±SEM. ^2^ Control (*n*=10), Treatment (*n*=10). ^a, b^ Bacterial abundance in the control and treatment groups belonging to the same sample collection site with different letters significantly differ (the Mann-Whitney U test, *P*<0.05).
