# Supplementary material for: Supplemental Clostridium butyricum MIYAIRI 588 Affects Intestinal Bacterial Composition of Finishing Pigs
Source: Microbes Environ. 2022 Sep 23;37(3):ME22011. doi: 10.1264/jsme2.ME22011 (PMC9530721; doi:10.1264/jsme2.ME22011)
Supplement: Supplementary file 1 — Supplementary Material [file 37_22011_s1.pdf]

Supplemental Table 1. Relative abundance (%) of *Clostridium_butyricum* derived from NGS analysis*, **

Group	Cecum	Colon	Feces
Control	1.63 ± 0.21	1.32 ± 0.29	3.10 ± 0.74
Treatment	2.07 ± 0.31	1.99 ± 0.40	2.74 ± 0.26

* Data are expressed as the mean ± SEM.

**Control (n=10), Treatment (n=10).
